# Antibiotic selection based on microbiology and resistance profiles of bile from gallbladder of patients with acute cholecystitis

**DOI:** 10.1038/s41598-021-82603-8

**Published:** 2021-02-03

**Authors:** Suk-Won Suh, Yoo Shin Choi, Seong-Ho Choi, Jae Hyuk Do, Hyoung-Chul Oh, Hong Jin Kim, Seung Eun Lee

**Affiliations:** 1 Department of Surgery, Chung-Ang University College of Medicine, Chung-Ang University Hospital, 224-1, Heuk Seok-Dong, Dongjak-Ku, Seoul, 156-755 South Korea; 2 Division of Infectious Diseases, Department of Internal Medicine, Chung-Ang University College of Medicine, Seoul, Korea; 3 Division of Gastroenterology, Department of Internal Medicine, Chung-Ang University College of Medicine, Seoul, Korea; 4 Department of Surgery, University of North Carolina At Chapel Hill, Chapel Hill, NC USA

**Keywords:** Gall bladder disease, Bacterial infection

## Abstract

With the progression of acute cholecystitis, antimicrobial therapy becomes important for infection control. Current antibiotic recommendations were mostly based on reports of patients with acute cholangitis whose bile specimens were sampled from the biliary tract. However, as most infections of acute cholecystitis are limited to the gallbladder, direct sampling from the site increases the probability of identifying the causative pathogen. We investigated 321 positive bile cultures from 931 patients with acute cholecystitis who underwent laparoscopic cholecystectomy between January 2003 and December 2017. The frequency of enterococci declined (P = 0.041), whereas that of Enterobacteriales (P = 0.005), particularly *Escherichia* (P = 0.008), increased over time. The incidence of ciprofloxacin-resistant Enterobacteriales showed a significant increasing trend (P = 0.031). Vancomycin-resistant *E.faecium*, carbapenem-resistant Enterobacteriales, and extended-spectrum beta-lactamase-producing Enterobacteriales were recently observed. In grade I and II acute cholecystitis, there were no significant differences in perioperative outcomes in patients with and without early appropriate antimicrobial therapy. In conclusion, the changing incidence of frequently isolated microorganisms and their antibiotic resistance over time would be considered before selecting antibiotics for the treatment of acute cholecystitis. Surgery might be a crucial component of infection control in grade I and II acute cholecystitis.

## Introduction

Most cases of acute cholecystitis result from the obstruction of the gallbladder outlet by an impacted gallstone, which causes an increase in intraluminal pressure, gallbladder distension, and wall edema and consequently progresses to gallbladder necrosis^1^. Bile is usually sterile during the early stages of acute cholecystitis and becomes infected as a secondary event. Previous studies have found bile to be infected in 9–42% of patients who underwent elective laparoscopic cholecystectomy, but the incidence of culture-positive bile increased to 35–65% of patients with acute cholecystitis^2,3^. Among patients with moderate to severe acute cholecystitis, antimicrobial therapy is important for limiting both the systemic septic response and local inflammation following cholecystectomy^4^. Appropriate antimicrobial therapy should be administered within 1 h for patients with septic shock and within 6 h of diagnosis for other less acutely ill patients^5^. However, results of bile culture cannot be obtained immediately after admission, and bile culture requires percutaneous puncture of the gallbladder. Therefore, an initial antimicrobial therapy is mainly based on the most effective empiric antibiotics reported in the literature.

The most commonly isolated microorganisms among pathogens in positive bile cultures are enterococci and bacteria from the order Enterobacteriales, such as *Escherichia* and *Klebsiella*. The empiric antibiotics recommended for these organisms are β-lactam-based/β-lactamase inhibitors, cephalosporins, carbapenems, and fluoroquinolones^6,7^. However, these choices are mostly based on previous studies of acute cholangitis, wherein bile specimens were sampled from the biliary tract using percutaneous transhepatic biliary drainage (PTBD) or endoscopic nasobiliary drainage (ENBD) and are potentially associated with microbial contamination from the catheter or by normal flora of the skin or gastrointestinal tract. The microbial and antibiotic resistance profiles of bile sampled from the gallbladder may provide more information in the context of acute cholecystitis because most such infections are limited to the gallbladder, and sampling directly from the infection site increases the likelihood of identifying the true causative pathogen. Medical environments have changed over time, resulting in changes in bile microbiologic and antibiotic resistance patterns. Therefore, it is important to collect data that inform early appropriate antimicrobial therapy for patients with acute cholecystitis.

This study aimed to investigate the trends of bacterial growth and antibiotic resistance patterns in the bile of gallbladder in patients with acute cholecystitis over time, to guide appropriate antibiotic recommendations. We also analyzed and compared the perioperative outcomes of patients who received and did not receive early appropriate antimicrobial therapy according to the grade of acute cholecystitis.

## Results

### Clinical characteristics of the patients

The mean age of the patients was 64.9 ± 14.1 years, and the number of males (52.6%) was slightly higher than that of females (47.4%) in this study. Most patients had acute calculous cholecystitis (93.1%) with grade II severity (81.3%). Among 94 patients (26.5%) who had combined common bile duct stone, endoscopic retrograde cholangiopancreatography (ERCP) was more frequently performed, than PTBD (17.7% vs. 8.7%). Initial laboratory findings showed elevated white blood cell (WBC) counts (12,780 ± 4,549 cells/µL) and total bilirubin (1.9 ± 1.9 mg/dL), direct bilirubin (1.1 ± 1.5 mg/dL), aspartate aminotransferase (AST, 150.9 ± 274.6 IU/L), alanine aminotransferase (ALT, 124.5 ± 201.3 IU/L), and alkaline phosphatase (ALP, 225.4 ± 215.5 IU/L) levels (Table 1).

**Table 1:** Clinical characteristics of patients.

Variable	n = 321
Age, mean (years)	64.9 ± 14.1
**Sex**	
Male	169 (52.6%)
Female	152 (47.4%)
**Cause of acute cholecystitis**	
Calculous	299 (93.1%)
Acalculous	22 (6.9%)
**Grade of acute cholecystitis**	
I	60 (18.7%)
II	261 (81.3%)
**Combined common bile duct stone**	94 (26.5%)
PTBD	31 (8.7%)
ERCP	63 (17.7%)
**Initial laboratory findings**	
WBC count cells/µL	12,780 ± 4,549
Total bilirubin (mg/dL)	1.9 ± 1.9
Direct bilirubin (mg/dL)	1.1 ± 1.5
AST (IU/L)	150.9 ± 274.6
ALT (IU/L)	124.5 ± 201.3
ALP (IU/L)	225.4 ± 215.5

Data are presented as the mean ± standard deviations or numbers with percentages in parentheses unless otherwise indicated.

*WBC* White blood cell, *AST* Aspartate transaminase, *ALT* Alanine transaminase, *ALP* Alkaline phosphatase.

### Frequencies of microorganisms isolated over time

**Figure 1:**
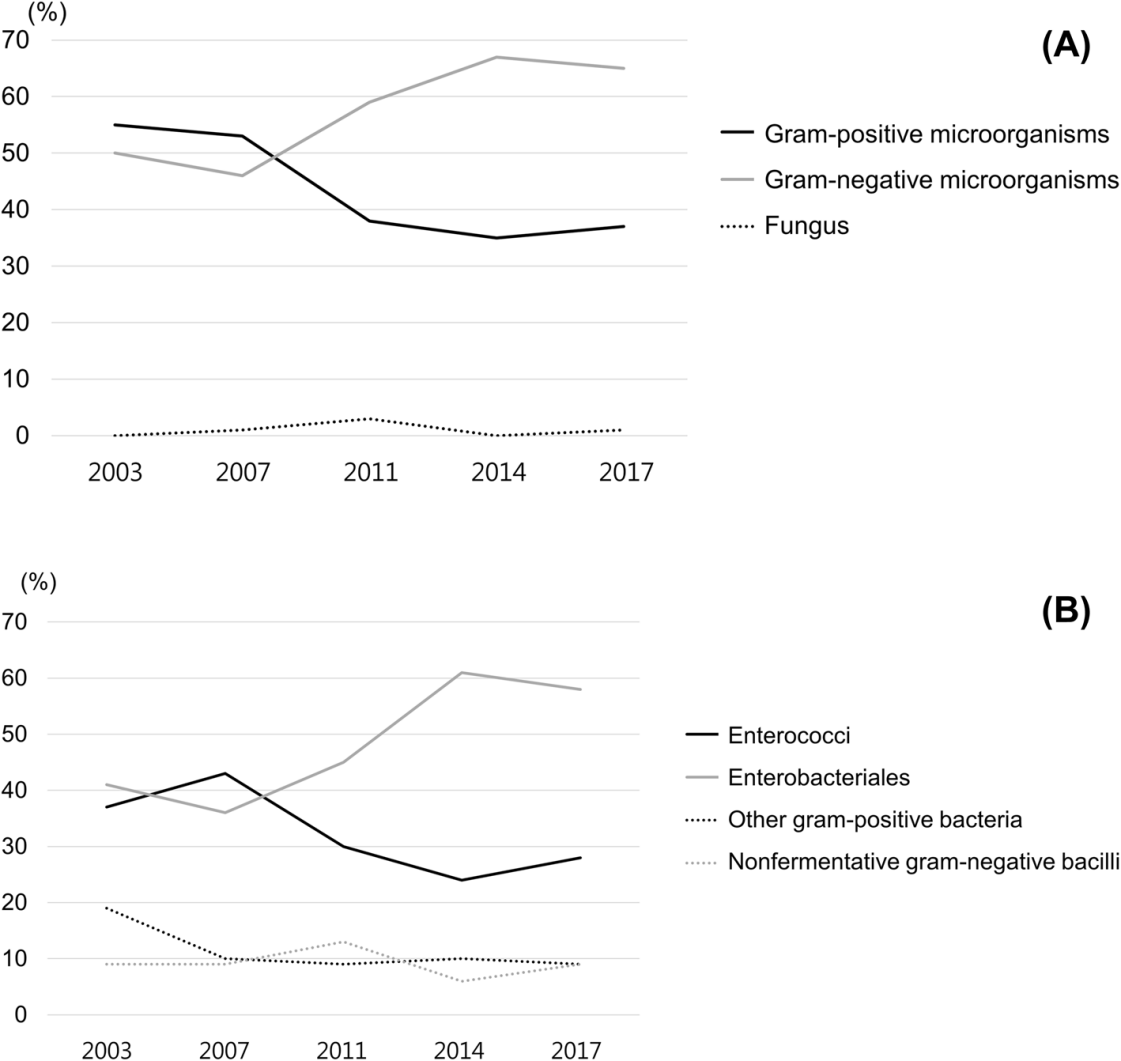
Trends in the frequencies of microorganisms isolated from the bile of gallbladder in acute cholecystitis patients between January 2003 and December 2017. (**A**) A significant decreasing trend in frequency was observed for gram-positive microorganisms (*P* = 0.016), whereas gram-negative microorganisms (*P* = 0.010) showed a significant increasing trend during the investigated period. There was no significant change in the frequency of fungal infections over time (*P* = 0.521). (**B**) Among the gram-positive microorganisms, the frequency of infections caused by enterococci (*P* = 0.041) significant declined over time, while there were no significant trends associated with infections caused by other types of gram-positive bacteria (*P* = 0.404). Among the gram-negative microorganisms, the frequency of infections caused by species from the order Enterobacteriales significantly increased over time (*P* = 0.005), but there was no significant trend associated with infections caused by non-fermentative bacilli (*P* = 0.606).

Most infections were apparently mono-microbial (98.1%) throughout the study period. The frequency of gram-positive microorganisms as the causative organisms of acute cholecystitis significantly declined over time (*P* = 0.016), decreasing from a frequency of 12 out of 22 (54.5%) between 2003 and 2005 to 45 out of 127 (35.4%) between 2015 and 2017. However, gram-negative microorganisms (*P* = 0.010) showed a significant increasing trend over the study period, rising from a frequency of 11 out of 22 (50.0%) between 2003 and 2005 to 83 out of 127 (65.4%) between 2015 and 2017. There was no significant change in the incidence of fungal infection over time (*P* = 0.521) (Fig. 1A). Among gram-positive microorganisms, although enterococci showed a significant decreasing trend over time (*P* = 0.041), there was no significant change in the incidence of other gram-positive microorganisms over time (*P* = 0.404). Among gram-negative microorganisms, there was a significant increase in the frequency of infections caused by members of the order Enterobacteriales over time (*P* = 0.005), but the incidence of non-fermentative, gram-negative bacilli showed no significant change over time (*P* = 0.606) (Fig. 1B).

### Trends of enterococci and Enterobacteriales isolates over time

**Figure 2:**
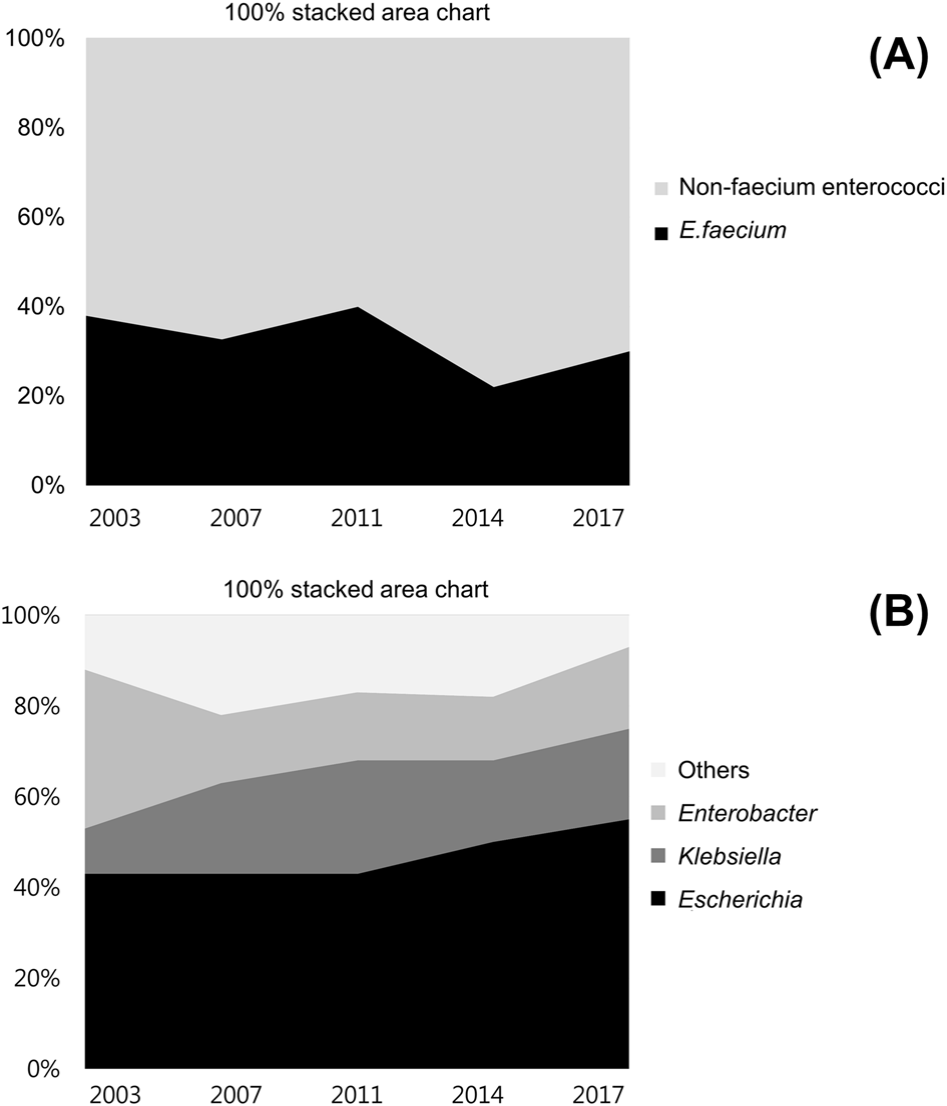
Relative proportions of enterococci and Enterobacteriales isolates obtained from the bile of gallbladder in acute cholecystitis patients over time. (**A**) The relative proportion of *E. faecium* isolates decreased over time, but there was no statistical significance (*P* = 0.207). (**B**) Among genera of the order Enterobacteriales, there was a significant increase in the relative proportion of *Escherichia* (*P* = 0.008), whereas there were no significant changes in the relative proportions of infections caused by *Klebsiella* (*P* = 0.212), *Enterobacter* (*P* = 0.637), or other Enterobacteriales (*P* = 0.162).

In terms of enterococci and Enterobacteriales isolates, the incidence of *E.faecium* decreased slightly over time (*P* = 0.207, Fig. 2A). A significant increase in frequency was observed for infections caused by *Escherichia* (*P* = 0.008), increasing from 4 out of 22 (18.2%) between 2003 and 2005 to 42 out of 127 (33.1%) between 2015 and 2017. However, the incidence of *Klebsiella* (*P* = 0.212) and *Enterobacter* (*P* = 0.637) infections showed no significant changes during the study period (Fig. 2B).

### Antibiotic resistance trends in association with enterococci and Enterobacteriales isolates over time

**Figure 3:**
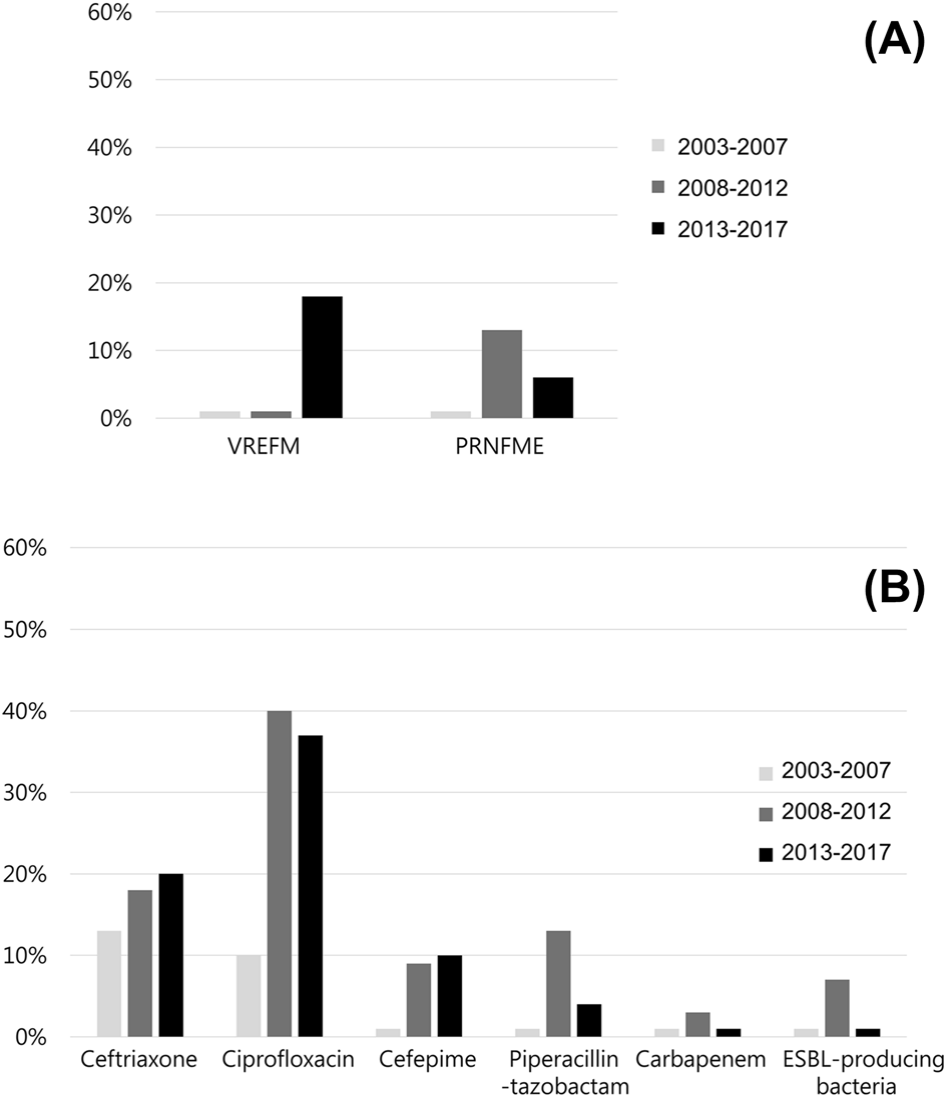
Antibiotic resistance trends in association with enterococci and Enterobacteriales isolates obtained from the bile of gallbladder in acute cholecystitis patients. (**A**) Vancomycin-resistant *E. faecium* (VREFM was first observed in the later period (2013–2017), but there was no statistical significance (*P* = 0.115), and there was also no significant trend in the proportion of penicillin-resistant non-*faecium* enterococci (PRNFME) (*P* = 0.546). (**B**) There was a significant increasing trend in the proportion of Enterobacteriales isolates that were resistant to ciprofloxacin (*P* = 0.031). The other investigated antibiotics were not associated with significant increase or decrease in the proportions of isolates that were resistant, but the proportion of isolates resistant to ceftriaxone continuously increased over time to a relatively high proportion (~ 20%) between 2013 and 2017. Notably, carbapenem-resistant Enterobacteriales (CRE) and extended-spectrum beta-lactamase (ESBL)-producing gram-negative bacteria were first observed between 2013 and 2017.

Temporal trends of antibiotic resistance against enterococci and Enterobacteriales obtained from the gallbladder of patients were analyzed. Vancomycin-resistant *E. faecium* (VREFM) was recently observed during the last 4 years of the study period, but the change in the proportion of VREFM over time was not statistically significant (*P* = 0.115). There was also no significant trend in the incidence of penicillin-resistant non-*faecium* enterococci (PRNFME) over time (*P* = 0.546, Fig. 3A). Among the Enterobacteriales isolates, the proportion of ciprofloxacin-resistant Enterobacteriales significantly increased over time, from 2 out of 20 (10.0%) between 2003 and 2007 to 37 out of 128 (35.9%) between 2013 and 2017 (*P* = 0.031). Other trends in the proportions of antibiotic-resistant isolates were not statistically significant, but the proportion of isolates resistant to ceftriaxone continuously increased over time, up to a relatively high incidence of about 20% between 2013 and 2017. Carbapenem-resistant Enterobacteriales (CRE) and extended-spectrum β-lactamase (ESBL)-producing gram-negative bacteria were first observed during the later years (2013–2017) of the period under investigation (Fig. 3B).

### Duration of preoperative antibiotic therapy and frequency of PTBD and ERCP over time

**Figure 4:**
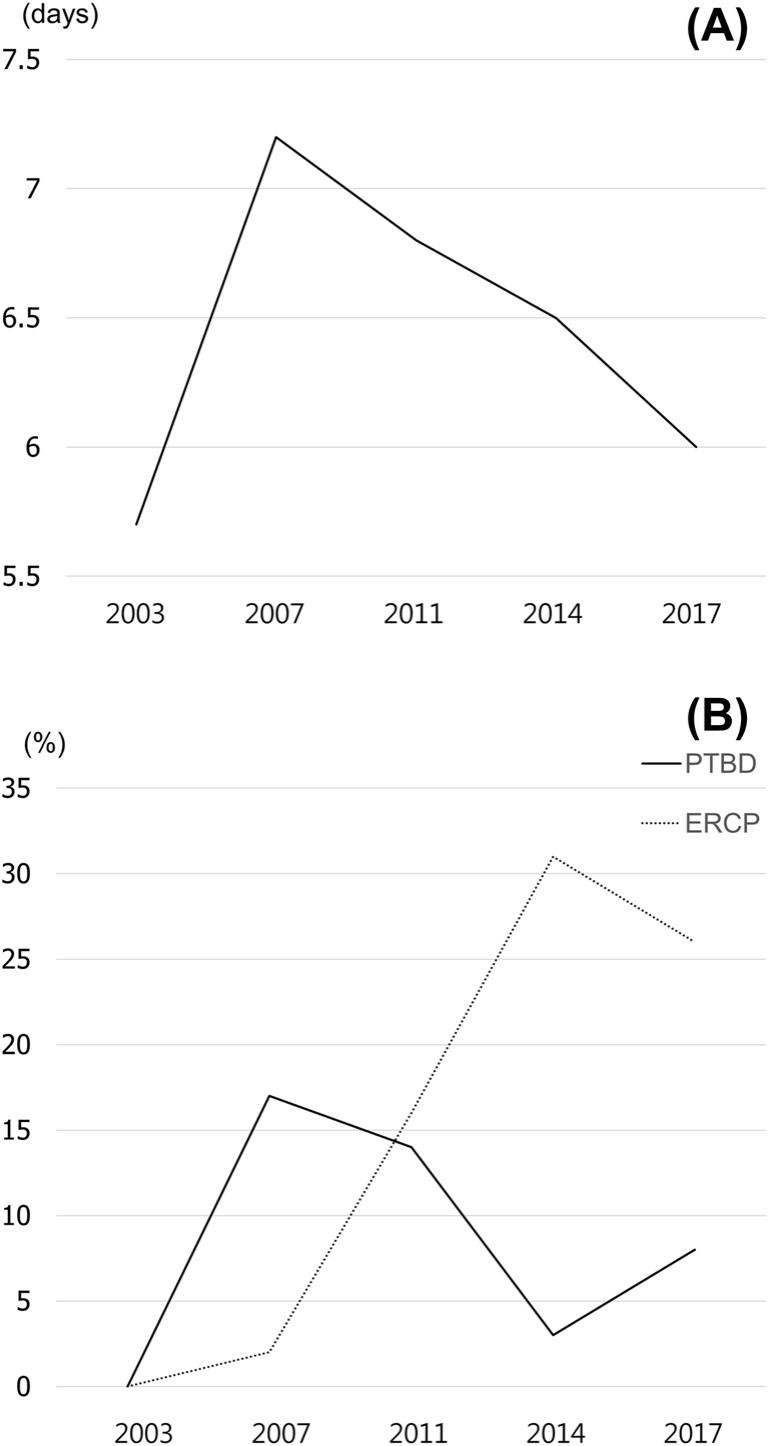
Duration of preoperative antibiotic therapy and frequency of PTBD and ERCP over time. (**A**) The duration of preoperative antibiotic therapy at initial period (2003–2005) was 5.7 ± 3.2, but increased to 7.2 ± 5.2 days between 2006 and 2008 and consistently decreased to 5.9 ± 6.0 days between 2015 and 2017, without statistical significance (*P* = 0.678). (**B**) The frequency of PTBD had a significant decreasing trend over time (*P* = 0.037), from 9 out of 53 (17.0%) between 2005 and 2007 to 2 out of 59 (3.4%) between 2012 and 2014, but increased in the recent period (11 of 131, 8.4%). Otherwise, ECRP had significant increasing trend over time (*P* = 0.000) from 1 out of 53 (1.9%) between 2005 and 2007 to 18 out of 59 (30.5%) between 2012 and 2014, but decreased in the recent period (34 of 131, 26.0%).

The duration of preoperative antibiotic therapy at initial period (2003–2005) was 5.7 ± 3.2, but increased to 7.2 ± 5.2 days between 2006 and 2008 and consistently decreased to 5.9 ± 6.0 days between 2015 and 2017, without statistical significance (*P* = 0.678, Fig. 4A). The frequency of PTBD had a significant decreasing trend over time (*P* = 0.037), from 9 out of 53 (17.0%) between 2005 and 2007 to 2 out of 59 (3.4%) between 2012 and 2014, but increased in the recent period (11 of 131, 8.4%). Otherwise, ECRP had significant increasing trend over time (*P* = 0.000) from 1 out of 53 (1.9%) between 2005 and 2007 to 18 out of 59 (30.5%) between 2012 and 2014, but decreased in the recent period (34 of 131, 26.0%) (Fig. 4B).

### Recent perioperative outcomes in grade I and II acute cholecystitis patients with and without early appropriate antimicrobial therapy

Over the last 4 years of the study period, we conducted a comparative analysis of perioperative outcomes based on the grade of acute cholecystitis according to the 2018 Tokyo Guidelines^8^ among patients who received early appropriate antimicrobial therapy and those who did not. Among patients with grade I acute cholecystitis, there were no significant differences in perioperative outcomes between patients who received early appropriate antibiotics and those who did not (Table 2). The same trend was observed among patients with grade II acute cholecystitis (Table 3). Among nine grade II acute cholecystitis patients with bacteremia, failure to administer early appropriate antimicrobial therapy (n = 6) was associated with longer operation times (90.8 ± 39.2 vs. 53.3 ± 27.5 min, *P* = 0.150), increased blood loss (376.7 ± 795.7 vs. 33.3 ± 15.3 mL, *P* = 0.339), a higher open conversion rate (33.3% vs. 0%, *P* = 0.257), and longer postoperative hospitalization (6.7 ± 4.5 vs. 5.0 ± 2.6 days, *P* = 0.507) compared with successful administration of early appropriate antimicrobial therapy (n = 3). However, none of these differences were statistically significant.

**Table 2:** Comparative analysis of the perioperative outcomes in grade I acute cholecystitis patients with and without early appropriate antimicrobial therapy in recent period.

Variable	With early appropriate antimicrobial therapy (n = 15)	Without early appropriate antimicrobial therapy (n = 8)	*P-*value
Age, mean (years)	63.9 ± 7.1	64.8 ± 7.5	0.814
**Sex**			
Male	8 (53.3%)	7 (87.5%)	0.101
Female	7 (46.7%)	1 (12.5%)	
Operation time (mins)	66.0 ± 44.1	81.9 ± 34.8	0.389
Estimated blood loss (mL)	60.7 ± 58.9	105.0 ± 162.4	0.347
Open conversion	1 (6.7%)	2 (25.0%)	0.214
Indwelling drain catheter	11 (73.3%)	7 (87.5%)	0.433
Wound infection	1 (6.7%)	0	0.455
Major postoperative complications	0	0	0.662
Postoperative hospital stay (days)	4.2 ± 2.1	4.4 ± 2.0	0.847

Data are presented as the mean ± standard deviations or numbers with percentages in parentheses unless otherwise indicated.

**Table 3:** Comparative analysis of the perioperative outcomes in grade II acute cholecystitis patients with and without early appropriate antimicrobial therapy.

Variable	With early appropriate antimicrobial therapy (n = 51)	Without early appropriate antimicrobial therapy (n = 40)	*P-*value
Age, mean (years)	67.0 ± 10.4	63.5 ± 13.7	0.223
**Sex**			
Male	32 (62.7%)	21 (52.5%)	0.325
Female	19 (37.3%)	19 (47.5%)	
Operation time (mins)	63.0 ± 29.3	61.6 ± 28.0	0.816
Estimated blood loss (mL)	66.0 ± 81.1	107.9 ± 317.0	0.366
Open conversion	2 (3.9%)	2 (5.0%)	0.803
Indwelling drain catheter	41 (80.4%)	27 (67.5%)	0.160
Wound infection	1 (2.0%)	2 (5.0%)	0.420
Major postoperative complications	1 (2.0%)	1 (2.5%)	0.862
Postoperative hospital stay (days)	6.5 ± 3.1	6.2 ± 2.7	0.616

Data are presented as the mean ± standard deviations or numbers with percentages in parentheses unless otherwise indicated.

## Discussion

Polymicrobial infections have been frequently reported in previous studies of biliary disease, and antibiotic regimens consisting of two or more agents are often recommended^9,10^. However, mono-microbial growth was predominant in this study, indicating that antibiotic selection should be different for patients with acute cholecystitis compared with those with other biliary tract infections.

Bile has antimicrobial properties, which results in the sterility of the biliary tract under non-pathogenic conditions^11^. However, gram-negative microorganisms, commonly found in the intestinal tract, such as Enterobacteriales were frequently isolated from patients with biliary infection^12^. Owing to the development of defense mechanisms, Enterobacteriales, especially *Escherichia*, became more resistant to bile than gram-positive microorganisms, thus becoming less sensitive to the deleterious effects of bile so that they frequently colonized the gallbladder and became an important reservoir for biliary infections^13,14^.

The proportion of infections caused by the gram-positive microorganisms including enterococci significantly declined over time, whereas the gram-negative microorganisms, especially Enterobacteriales are becoming more prevalent, and most commonly isolated among patients with acute cholecystitis in this study. There might have several reasons for these changes. At first, it might be associated with the recent extensive use of oral fluoroquinolone for urinary tract infections, pneumonia, and skin or soft tissue infections in the community. Although enterococci were intrinsically less susceptible to fluoroquinolones, gut microbiota, specifically enterococcal populations, were highly impacted by ciprofloxacin, with a decrease observed in their density and diversity in healthy volunteers^15^. Second, the duration of preoperative antibiotic therapy would be related with these changes because most of recommended antibiotics for biliary infection were susceptible to Enterobacteriales other than enterococci. In this study, the duration of preoperative antibiotic therapy was consistently decreased from the second period to the recent period. Third, the frequency of biliary intervention either PTBD or ERCP, performed for patients with combined common bile duct stone would be associated with these changes. A previous study had shown that enterococci were commonly isolated from the bile of patients with stents or from those who had undergone PTBD^16^. In contrast, patients who underwent ECRP might have more chances of ascending infection from the microorganisms of intestinal tract, as Enterobacteriales. In this study, the frequency of PTBD had a significant decreasing trend, whereas ECRP had significant increasing trend over time. However, the frequency of those procedures were reversed in the recent period that it might be a reason for a recent resurgence of gram-positive microorganisms, including enterococci in this study.

Broad-spectrum β-lactam and β-lactamase inhibitors, such as ampicillin-sulbactam, have been recommended as the first-line drugs to treat enterococcal infections^8^. Although penicillin and ampicillin provide good coverage against non-*faecium* enterococci, such as *E. faecalis*, *E. gallinarum*, *E. casseliflavus*, and *E. avium*, enterococcal resistance to these antibiotics increased considerably over time during the study period. *E. faecium* has been reported to be more resistant to antibiotics than non-*faecium* enterococci^7^, based on evidence of commonly used antibiotics, including ampicillin-sulbactam, being ineffective against *E. faecium*. Therefore, vancomycin is recommended for infections with *E. faecium*^7^. The first detection of VREFM in this study was from samples obtained during the later years (2013–2017) of the investigated period, and nearly all of these patients had grade II acute cholecystitis (94.7%). Hence, other antibiotics, including linezolid and tigecycline, which provide good coverage against VREFM, should be considered for patients with such advanced infections. Although one report noted the poor effectiveness of tigecycline for severely ill patients with septic shock^17^, tigecycline can be used in several other cases because of its broad spectrum of effectiveness against gram-negative microorganisms, including ESBL-producing bacteria. As enterococci have seldom been associated with bacteremia, it is still controversial to administer antibiotics when enterococci are isolated from culture samples. However, antimicrobial therapy should be strongly considered for high-risk patients such as immunocompromised patients with nosocomial infections, severely ill patients with a history of taking broad-spectrum antibiotics, and patients at high risk of endocarditis^18,19^.

For gram-negative microorganisms, piperacillin-tazobactam and third- or fourth-generation cephalosporins are recommended as the first drugs of choice, and fluoroquinolones and carbapenems are recommended as the second choice, depending on the severity of the illness and antimicrobial susceptibility patterns^8^. However, this study has demonstrated a significant increasing frequency of ciprofloxacin-resistant Enterobacteriales infections, likely, at least partly, because of its extensive usage in the community. Additionally, nearly 20% of causative bacteria were resistant to ceftriaxone during the recent period of the study. Therefore, these antibiotics are not appropriate for initial empiric antimicrobial therapy, and piperacillin-tazobactam or cefepime, which have broader spectra and lower resistance rates, would be more appropriate, especially for patients with severe infections. For patients who have been recently exposed to serial antimicrobial therapy, carbapenem or tigecycline should be used, considering the possibility of infection with ESBL-producing gram-negative bacteria. CRE species have emerged as important healthcare-associated pathogens because of extensive drug resistance and associated high morbidity and mortality rates^20^. One case of CRE was detected in this study; continuous monitoring for these bacteria is required.

The role of antimicrobial therapy varies depending on the severity of the illness and etiologic characteristics. In grade I acute cholecystitis, as it is not obvious whether bacteria play a significant role, antimicrobial therapy is administered to prevent progression to infection before cholecystectomy. For grade II acute cholecystitis, antimicrobial therapy is therapeutic and required until the gallbladder is removed^8,21^. In this study, in contrast to patients with grade I and II acute cholecystitis, bacteremia, or VREFM infection, patients who did not receive early appropriate antibiotics did not require additional antibiotic treatment or postoperative hospitalization. Thus, surgery might be more important for the treatment of grade I and II acute cholecystitis, even as an infection control measure. Although the analysis of patients with bacteremia did not reach statistical significance, because of the small number of patients in this study, early appropriate antimicrobial therapy for those patients seems to be important as we observed marked differences in operation times, estimated blood loss, open conversion rate, and postoperative hospitalization duration according to the bacteremia status. Most patients with bacteremia might have clinical deterioration and can be classified as grade III acute cholecystitis and are therefore not suitable for surgery. Hence, only a small number of those patients, who were in relatively good condition, were included in this study.

This study has several limitations. First, this was a retrospective study because of which the accuracy of the data analyzed relied on the completeness of the medical records. Second, because of differences in bacterial growth and other parameters, more experimental evidence would be needed to determine or confirm antibiotic resistance in the future. Furthermore, over time, there were changes in the list of antibiotic resistance tests available at our hospital, rendering the data on some antibiotics to be insufficient for analysis. Finally, the results of our microbiological and antibiotic resistance analyses may be different because of regional and institutional differences.

In conclusion, the incidence of frequently isolated microorganisms and their antibiotic resistance profiles changed over time. The frequency of infections caused by gram-positive microorganisms, including enterococci, significantly declined during the period under study, whereas infections caused by gram-negative microorganisms, including Enterobacteriales, especially *Escherichia*, showed a significant increasing trend. Of the antibiotics previously recommended for acute cholecystitis, ciprofloxacin and ceftriaxone were among the agents that were no longer as effective and hence, are inappropriate for initial empiric antimicrobial therapy. VREFM, CRE, and ESBL-producing bacteria were first detected during the later years of the period under study; continuous monitoring of these resistant microorganisms is required. For grade I and II acute cholecystitis, surgery might be crucial for treatment and infection control.

## Materials and methods

We retrospectively reviewed 321 positive bile cultures from 931 patients who underwent cholecystectomy for acute cholecystitis between January 2003 and December 2017 at our hospital. The diagnostic criteria and severity assessment criteria for acute cholecystitis provided by the 2018 Tokyo Guidelines^8^ were used. The following clinical and demographic data were captured for all patients: age, sex, cause of acute cholecystitis (calculous or acalculous), grade of acute cholecystitis, and initial laboratory findings (WBC count, total bilirubin, direct bilirubin, AST, ALT, and ALP). We captured the overall incidence of the detected microbes—including multidrug-resistant pathogens, such as ESBL-producing gram-negative bacteria, CRE, and VREFM—and their antibiotic susceptibility patterns. To validate the clinical significance of early and appropriate antimicrobial therapy by acute cholecystitis grade, we compared the perioperative outcomes (operation time, estimated blood loss, open conversion rate, indwelling drain catheter insertion rate, presence of wound infection, major postoperative complications [classified according to a modified version of the original Clavien system], duration of preoperative antibiotics, and postoperative hospital stay) of patients who received and did not receive early appropriate antimicrobial therapy.

This study was approved by the institutional review board of Chung-Ang University Hospital, Seoul, Korea (CAUH IRB No. 1911-016-16289). As this was a retrospective cohort study, the requirement for written informed consent was waived by the institutional review board. All methods were carried out in accordance with relevant guidelines and regulations.

### Cultures and antibiotic susceptibility testing

Bile was swabbed in an aseptic manner for microbial assessment immediately after intraoperative retrieval of the gallbladder specimen. The specimens were inoculated on blood agar, chocolate agar, and MacConkey agar. The plates were cultivated at 37 °C in a 50 mL/L CO2 aerobic chamber, inoculated on phenylethanol blood agar, and cultivated for 48 h in a Forma anaerobic chamber (Forma Scientific, Marietta, OH, USA). All colonies that formed after 48 h were identified and tested for antibiotic susceptibility using the VITEK2 system (bioMérieux Vitek Inc., Durham, NC, USA). Antibiotic susceptibilities were reported as follows: susceptible (S) meant that poor bacterial growth was detected with adequate antibiotics; indeterminate (I) meant that an inappropriate antibiotic was used; and resistant (R) meant that the bacterial colony continued to grow despite the presence of a normally effective antibiotic.

### Statistical analysis

For intergroup comparisons, the distribution of data was first evaluated for normality using the Shapiro–Wilk test. Normally distributed data are presented here as means ± standard deviations, and between-group comparisons were conducted using Student’s t-test or Kruskal–Wallis test. Between-group comparisons of descriptive data were conducted using the χ^2^ test and Fisher’s exact test. The test for trend was performed by linear-by-linear association. Statistical analyses were conducted using SPSS Statistics for Windows, version 19.0 (IBM Corp., Armonk, NY, USA).

## Data Availability

All data needed to evaluate the conclusions of the study are present in the paper.

## Abbreviations

PTBD: Percutaneous trans-hepatic biliary drainage
ENBD: Endoscopic nasobiliary drainage
MDR: Multidrug-resistant
ESBL: Extended-spectrum beta-lactamase
CRE: Carbapenem-resistant Enterobacteriales
VRE: Vancomycin-resistant enterococci
TB: Total bilirubin
DB: Direct bilirubin
AST: Aspartate aminotransferase
ALT: Alanine aminotransferase
ALP: Alkaline phosphatase
